# iHam and pyHam: visualizing and processing hierarchical orthologous groups

**DOI:** 10.1093/bioinformatics/bty994

**Published:** 2018-12-03

**Authors:** Clément-Marie Train, Miguel Pignatelli, Adrian Altenhoff, Christophe Dessimoz

**Affiliations:** 1 SIB Swiss Institute of Bioinformatics, Lausanne, Switzerland; 2 ETH Zurich, Department of Computer Science, Zurich, Switzerland; 3 Center for Integrative Genomics, University of Lausanne, Lausanne, Switzerland; 4 Open Targets; 5 EMBL-European Bioinformatics Institute, Wellcome Genome Campus, Hinxton, Cambridge, UK; 6 Department of Computational Biology, University of Lausanne, Lausanne, Switzerland; 7 Department of Genetics, Evolution and Environment, University College London, London, UK; 8 Department of Computer Science, University College London, London, UK

## Abstract

**Summary:**

The evolutionary history of gene families can be complex due to duplications and losses. This complexity is compounded by the large number of genomes simultaneously considered in contemporary comparative genomic analyses. As provided by several orthology databases, hierarchical orthologous groups (HOGs) are sets of genes that are inferred to have descended from a common ancestral gene within a species clade. This implies that the set of HOGs defined for a particular clade correspond to the ancestral genes found in its last common ancestor. Furthermore, by keeping track of HOG composition along the species tree, it is possible to infer the emergence, duplications and losses of genes within a gene family of interest. However, the lack of tools to manipulate and analyse HOGs has made it difficult to extract, display and interpret this type of information. To address this, we introduce **i**nteractive **H**OG **a**nalysis **m**ethod, an interactive JavaScript widget to visualize and explore gene family history encoded in HOGs and **py**thon **H**OG **a**nalysis **m**ethod, a python library for programmatic processing of genes families. These complementary open source tools greatly ease adoption of HOGs as a scalable and interpretable concept to relate genes across multiple species.

**Availability and implementation:**

iHam’s code is available at https://github.com/DessimozLab/iHam or can be loaded dynamically. pyHam’s code is available at https://github.com/DessimozLab/pyHam and or via the pip package ‘pyham’.

## 1 Introduction

The evolution of a gene family describes the history of all the genes that shared a common ancestral gene. Those genes called homologs can be distinguished into orthologs if they start diverging by speciation and paralogs if they start diverging by duplication (Fitch, 1970). In comparative genomics, gene families are a fundamental resource since they tend to represent the links between several organisms from a gene centric perspective and allow us to understand how genes and genomes have evolved over time.

The evolutionary history of gene families can be studied by visualizing reconciled gene trees, using web-based resources such as Ensembl (Herrero *et al.*, 2016), HOGENOM/HOVERGEN (Dufayard *et al.*, 2005), EggNOG (Huerta-Cepas *et al.*, 2016), PhylomeDB (Huerta-Cepas *et al.*, 2014) or tools such as ETE (Huerta-Cepas *et al.*, 2010) and SylvX (Chevenet *et al.*, 2016). However, when considering large families across many species, reconciled gene trees can become prohibitively complex to infer and interpret.

As a scalable alternative to reconciled gene trees, the concept of Hierarchical Orthologous Groups (HOGs) is increasingly adopted. HOGs generalize Fitch’s definition of orthology to more than two species, by grouping sequences that have descended from a common ancestral gene within a clade of interest. Thus, the set of all HOGs defined for a given clade corresponds to the set of ancestral genes in the common ancestor of that clade. Furthermore, if HOGs are available for nested clades (e.g. vertebrates versus mammals), the difference between their HOG repertoires imply gene duplication and loss events on the branch separating them: a HOG split implies a duplication, while a HOG disappearance implies a loss.

HOGs are inferred by several leading orthology databases such as OrthoDB (Zdobnov et al., 2017), EggNOG (Huerta-Cepas *et al.*, 2016), HieranoidDB (Kaduk *et al.*, 2017) or OMA (Altenhoff *et al.*, 2018). In OMA, for instance, some HOGs connect large gene families of over 100 000 members across 1000’s of genomes. Because of this complexity, manual exploration of gene families encoded in HOGs can be challenging. As of now, there is a lack of tool for visualizing, exploring and processing HOGs to tackle specific biological questions.

In this application note, we introduce two tools to facilitate the visualization and analysis of HOGs: **i**nteractive **H**OG **a**nalysis **m**ethod (iHam) for web-based interactive visualization and exploration of individual HOGs and **py**thon **H**OG **a**nalysis **m**ethod (pyHam) to perform aggregate analyses.

## 2 iHam

**Fig. 1.**
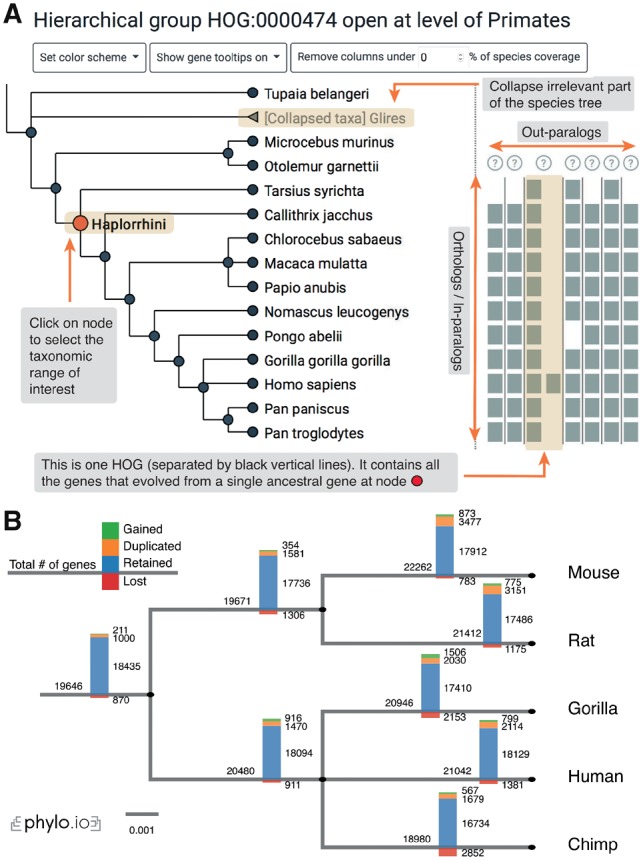
(**A**) iHam. An excerpt of the Tetraspanin family at the Haplorrhini level: the tree depicts relationships between species, squares depict genes and HOGs are delineated by vertical bars. (**B**) pyHam can be used to map gene losses, duplications or new appearances (‘gained’) onto species trees (here, using the NCBI taxonomy tree)

iHam is an interactive JavaScript tool to visualize the evolutionary history of a specific gene family encoded in HOGs. The viewer is composed of two panels (Fig. 1A): a species tree which lets the user select a node to focus on a particular taxonomic range of interest, and a matrix that organizes extant genes according to their membership in species (rows) and HOGs (columns). The tree-guided matrix representation of HOGs facilitates: (i) to delineate orthologous groups at given taxonomic ranges, (ii) to infer duplication and loss events in the species tree, (iii) gauge the cumulative effect of duplications and losses on gene repertoires and (iv) to identify potential mistakes in genome assembly, annotation or orthology inference (e.g. if losses are concentrated on terminal branches—suggestive of incomplete genomes; or if the species coverage within a HOG looks implausible—suggestive of orthology inference error).

Users can customize the view in different ways. They can color genes according to protein length or GC-content. Low-confidence HOGs can be masked. Irrelevant species clades can be collapsed. iHam is a reusable web widget that can be easily embedded into a website; for instance, it is used to display HOGs in OMA (http://omabrowser.org; Altenhoff *et al.*, 2018). Implemented as a JavaScript library using the TnT framework (Pignatelli, 2016), iHam merely requires as input HOGs in the standard OrthoXML format (Schmitt *et al.*, 2011) and the underlying species tree in newick or PhyloXML format (supported resources listed in Table 1).

**Table:** Table **1.** Support for iHam and pyHam by various HOG inference resources

Resource	Species tree format	OrthoXML	iHam Support	pyHam Support
OMA browser	PhyloXML and Newick	All HOGs, or one HOG at a time	YES	YES
OMA standalone	PhyloXML and Newick	All HOGs	YES	YES
Ensembl	Newick	One HOG at a time	YES	YES
HieranoidDB	Newick	One HOG at a time	YES	YES

## 3 pyHam

pyHam makes it possible to extract useful information from HOGs encoded in standard OrthoXML format. It is available both as a python library and as a set of command-line scripts. Input HOGs in OrthoXML format are available from multiple bioinformatics resources, including OMA, Ensembl and HieranoidDB (Table 1).

The main features of pyHam are: (i) given a clade of interest, extract all the relevant HOGs, each of which ideally corresponds to a distinct ancestral gene in the last common ancestor of the clade; (ii) given a branch on the species tree, report the HOGs that duplicated on the branch, got lost on the branch, first appeared on that branch or were simply retained; (iii) repeat the previous point along the entire species tree and plot an overview of the gene evolution dynamics along the tree (Fig. 1B) and (iv) given a set of nested HOGs for a specific gene family of interest, generate a local iHam web page to visualize its evolutionary history.

## 4 Conclusion

The combination of iHam and pyHam enable users to unlock the full potential of HOGs.
